# Individual and Synergistic Relationships of Low Muscle Mass and Low Muscle Function with Depressive Symptoms in Korean Older Adults

**DOI:** 10.3390/ijerph181910129

**Published:** 2021-09-27

**Authors:** Youngyun Jin, Seamon Kang, Hyunsik Kang

**Affiliations:** College of Sport Science, Sungkyunkwan University, Suwon 16419, Korea; player53@skku.edu (Y.J.); abtkang2@gmail.com (S.K.)

**Keywords:** sarcopenia, physical performance, depression, Korean older adults

## Abstract

This study examined the relationship of low appendicular skeletal muscle mass and low muscle function with depressive symptoms in Korean older adults. Community-dwelling Korean older adults aged 65 years and older (*n* = 521) participated in this study. Appendicular muscle mass (ASM) and muscle function (MF) scores were assessed using dual-energy X-ray absorptiometry (DXA) scanning and sit-to-stand mean power based on a 30 s chair stand test, respectively. Depressive symptoms were evaluated using the Korean form of the Center for Epidemiologic Studies Depression Scale. Logistic regression was used to estimate the odds ratios (ORs) and 95% confidence intervals (CIs) of depressive symptoms according to ASM- and MF-based subgroups; normal ASM/normal MF, low ASM/normal MF, normal ASM/low MF, and low ASM/low MF. The prevalence of depressive symptoms was 21.3% in all patients: 20.7% in women and 21.5% in men. Compared to the normal ASM/normal MF participants (OR = 1), the risk of depressive symptoms rose incrementally in subjects with low ASM/normal MF (OR = 2.963, *p* = 0.019), normal AMS/low MF (OR = 3.843, *p* = 0.002), and low ASM/low MF (OR = 7.907, *p* < 0.001), respectively. The current findings suggest that the coexistence of low ASM and low MF is significantly and independently associated with an increased risk for depressive symptoms, with dynapenia having a stronger relationship.

## 1. Introduction

Depression is a common illness associated with serious physical and psychological problems worldwide. As of 2017, it was estimated that approximately 1 million people worldwide suffered from depression [1]. In South Korea, the prevalence of depression is about 21% in the older population and is steadily rising. The total economic burden of depression was estimated to be USD 4.049 million in 2005 [2]. By 2012, this figure had dramatically increased to USD 1.331 billion [3].

Etiologically, lifestyle risk factors, health conditions, and low socioeconomic status are well-known risk factors for depression and/or depressive symptoms. Additionally, a relationship between depression and sarcopenia also exists in the literature [4]. In fact, sarcopenia leads to serious physical and psychological health problems of physical disability, depression, and suicidal ideation in geriatric populations [5].

The Asian Working Group for Sarcopenia (AWGS) defines sarcopenia as having poor physical performance and/or low muscle strength in addition to low muscle mass [6]. Pathologically, the loss of muscle strength, so-called dynapenia [7], precedes the loss of muscle mass to a greater degree [8,9], indicating that the former, rather than the latter, may be more involved in declines in physical function and adverse health outcomes in older adults [10].

In a clinical setting, low handgrip strength was found to be significantly associated with an increased risk of depressive symptoms, along with poor cognitive function and quality of life in older adults [11]. Previous studies involving Korean populations showed that depression was significantly associated with low skeletal muscle mass [12] or low handgrip strength [13]. However, findings from previous studies have not been consistent in Korean populations. For example, dual-energy X-ray absorptiometry (DXA)-based low muscle mass was not associated with the prevalence of depression or depressed symptoms V [14]. By contrast, late-life depression was significantly associated with DXA-based low muscle mass [15] or handgrip strength [16]. Although this finding has not been completely explained, the definition of sarcopenia may explain some of the inconsistency in this relationship.

To the best of our knowledge, however, the individual and synergistic role of low appendicular skeletal muscle (ASM) and low muscle function (MF) in determining the risk for depression is poorly understood, especially in Korean older adults. We hypothesized that both low ASM and low MF are significantly associated with increased depression risk in older adults, with low MF rather than low ASM playing a dominant role in the relationship. This study examined the individual and synergistic relationships of low ASM and low MF with depressive symptoms in Korean older adults.

## 2. Materials and Methods

### 2.1. Subjects

**Figure 1:**
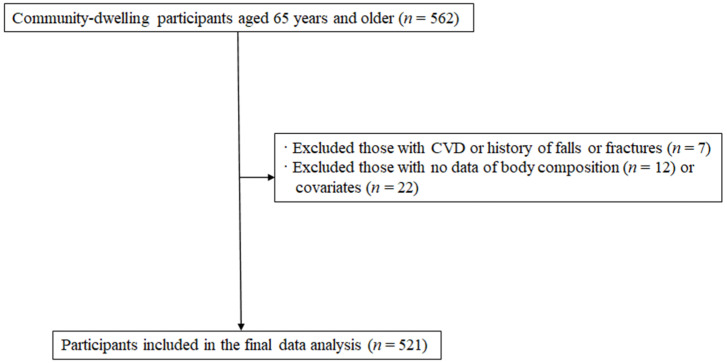
Flowchart of the study participants.

Figure 1 presents a flowchart of study participants. In a cross-sectional study design, we initially recruited 562 participants aged 65 years and older (mean age 74.1 ± 6.2 years) from local community centers in the city of Suwon, Republic of Korea, between February 2017 and October 2019. We then excluded the following: people who had CVD or a history of recurrent falls or fractures in previous years (*n* = 7) or no DXA-based body composition data (*n* = 12). Additionally, we also excluded those who had no data of covariates (*n* = 22). The remaining 521 participants were included in the final data analyses. Sample size was calculated on the basis of our preliminary study. Using an expected correlation coefficient of 0.14, we calculated that a sample size of 540 will be required at an α level of 0.05 and β level of 0.85, with the assumption of approximately 20% dropout rate (G*Power v3.1). The appropriate institutional review board reviewed and approved the study (approval no. SKKU 2018-06-005-003) in accordance with the Declaration of Helsinki. Written informed consent was obtained from all participants prior to their participation.

### 2.2. Outcomes

#### 2.2.1. Determination of Whole Body Composition

Whole body composition, including height, weight, muscle mass, and body fat, was assessed using a DXA (QDR 4500A, Hologic, Waltham, MA, USA) according to a previously validated procedure [17]. Waist circumference (WC) was taken at the midpoint between the bottom of the ribcage and the top of the iliac crest. Body mass index (BMI) was calculated as body weight (kg) divided by height (m^2^).

#### 2.2.2. Determination of Sarcopenia

Sarcopenia is defined according to the AWGS consensus report [6]. ASM was calculated as the sum of DXA-based muscle mass in arms and legs [18]. Sarcopenic index (SI) was then calculated as total ASM (kg) divided by height (kg/m^2^) according to the 2019 AWGS consensus updates on sarcopenia diagnosis and treatment [19], where the cut-off value of SI for low ASM was <7.0 kg/m^2^ for men and <5.4 kg/m^2^ for women.

#### 2.2.3. Sit and Stand Muscle Power Test

Assessment of muscle power as an index of MF was conducted using the 30 s chair stand test of which its reliability and validity were previously tested and reported in older adults [20]. In our laboratory, we routinely performed the 30 s chair stand test for older adults, with intraclass correlation coefficient of 0.890 (*p* < 0.001) and test–retest intra-rater reliability of 0.957 (*p* < 0.001).

After 10 min of warm-up and a demonstration by the tester, a practice trial of one repetition was allowed to check proper form, followed by the 30 s test trial. In brief, participants were seated in the middle of a chair without arms (43.2 cm) and back straight, with arms crossed at the wrist and held against the chest, and feet approximately a shoulder width apart. On command, they stood fully upright and then sat back down. The participant was encouraged to complete as many full stands as possible within 30 s, and the total number of repetitions was recorded. Then, sit and stand muscle power was calculated using a previously validated equation [21,22].

Relative muscle power (W·kg^−1^) was calculated as sit and stand mean muscle power (W) normalized to total body mass (kg). The cut-points for low relative MF used in the current study were 2.6 W·kg^−1^ and 2.16 W·kg^−1^ for men and women, respectively, which were recently tested and validated in a sample of 9320 older adults aged 60–103 years [23].

#### 2.2.4. Determination of Depressive Symptoms

Depressive symptoms were assessed with the 20-item Center for Epidemiologic Studies Depression Scale (CES-D) via a face-to-face interview that was conducted by a trained physician [24]. A score of ≥16 was indicative of depressive symptoms [25].

#### 2.2.5. Covariates

Age, education (years), alcohol consumption, smoking, and number of prescribed medications were assessed using an interviewer-administered questionnaire. Self-reported number of prescribed medications for diabetes, hypertension, hyperlipidemia, osteoarthritis, cardiac disease, or cancer were assessed. Alcohol consumption (i.e., ≤1 drink per week vs. ≥2 per week) and smoking status (i.e., past/current smoking vs. no smoking) were also reported.

The participants were asked about the frequency (times/week) and duration (minutes) of their daily physical activity (PA) by intensity (light, moderate, vigorous) using the Korean version of the International Physical Activity Questionnaire short form (IPAQ-SF). Total weekly PA (MET-min/week) was calculated by summing the products of duration by intensity for each PA type (walking = 3.3 METs, moderate PA = 4.0 METs, vigorous PA = 8.0 METs).

Cognitive function was measured using the Korean Mini-Mental State Examination (K-MMSE), which was tested and validated in Korean populations [26]. The 2.44 m up-and-go test to assess agility (seconds) was performed according to a previously validated protocol [20]. Handgrip strength of participants’ dominant hand was measured using a handgrip dynamometer (TANITA No. 6103, Tokyo, Japan). Each participant performed two trials with 1 min pause between trial, with verbal encouragement during each trial. The best value of two trials was recorded as the score for voluntary maximal handgrip strength (kg).

### 2.3. Statistical Analysis

Prior to statistical analyses, normality of data distribution was confirmed with QQ plotting. Outcomes of descriptive statistics are presented as means ± standard deviations. Descriptive statistics were performed with analysis of variance and chi-square test for continuous and categorical variables, respectively, which are presented as the mean ± standard deviation (SD) and number (n) or percentage (%), respectively. To test the research hypothesis, study participants were classified into four subgroups based on the ASM and MF criteria as mentioned above (i.e., normal ASM/normal MF, low ASM/normal MF, normal ASM/low MF, low ASM and low MF). In addition, study participants were also dichotomized as not having depressive symptoms or having depressive symptoms, based on the cut-point of 16 or higher CES-D score as mentioned above. Multivariate logistic regression was used to estimate odds ratio (OR) and 95% confidence intervals (95% CI) for depressive symptoms according to the ASM- and MF-based subgroups. All statistical analyses were conducted using the SPSS statistical software, version 21.0 (SPSS Inc., Chicago, IL, USA). Statistical significance was tested at *p* = 0.05.

## 3. Results

Table 1 represents descriptive statistics of the study participants. The prevalence of depressive symptoms was 21.3% in total, 20.7% in men, and 21.5% in women. Men had lower BMI (*p* = 0.035), lower waist circumference (*p* < 0.001), and lower number of medications (*p* < 0.001) in conjunction with more years of education (*p* < 0.001), greater alcohol consumption (*p* < 0.001), and higher smoking rate (*p* < 0.001) than women. Men also had higher ASM (*p* < 0.001), greater handgrip strength (*p* < 0.001), better time up-and-go test scores (*p* < 0.001), better 30 s chair stand test scores (*p* < 0.001), higher absolute and relative muscle power (*p* < 0.001 and *p* < 0.001, respectively), higher physical activity (*p* < 0.001), and higher MMSE-DS scores (*p* < 0.001) than women. No gender difference was found in mean age, WC, and CED-S scores between men and women.

Table 2 compares outcomes of measured variables among ASM- and MF-based subgroups. With respect to sociodemographics and body composition, there were significant group differences in mean age, education, BMI, and WC among ASM- and MF-based subgroups. Specifically, individuals with low ASM only (*p* < 0.001) or low MF only (*p* < 0.001) or low ASM plus low MF (*p* < 0.001) were older compared to individuals with normal ASM plus normal MF. Individuals with low MF only or low ASM plus low MF had lower educational background (*p* < 0.001 and *p* < 0.001, respectively) and lower BMI (*p* < 0.001 and *p* = 0.003, respectively) compared to individuals with normal ASM plus normal MF. In addition, individuals with low MF only had a higher WC (*p* = 0.035) compared to individuals with normal ASM and normal MF.

With respect to parameters of physical performance test, depressive symptoms, and cognitive function, there were significant group differences in handgrip strength, time up-and-go test, 30 s sit-to-stand test, sit-to-stand power, CES-D, and MMSE-DS among ASM- and MF-based subgroups. Specifically, individuals with low MF only or low ASM plus low MF had lower handgrip strength (*p* < 0.001 and *p* < 0.001, respectively), lower time up-and-go test performance (*p* < 0.001 and *p* < 0.001, respectively), lower 30 s sit-to-stand test performance (*p* < 0.001 and *p* < 0.001, respectively), and lower sit-to-stand power (*p* < 0.001 and *p* < 0.001, respectively) compared to individuals with normal ASM plus normal MF. In addition, individuals with low MF only or low ASM plus low MF had higher CES-D scores (*p* < 0.001 and *p* < 0.001, respectively) and lower MMSE-DS scores (*p* < 0.001 and *p* = 0.048, respectively) compared to individuals with normal ASM plus normal MF.

**Figure 2:**
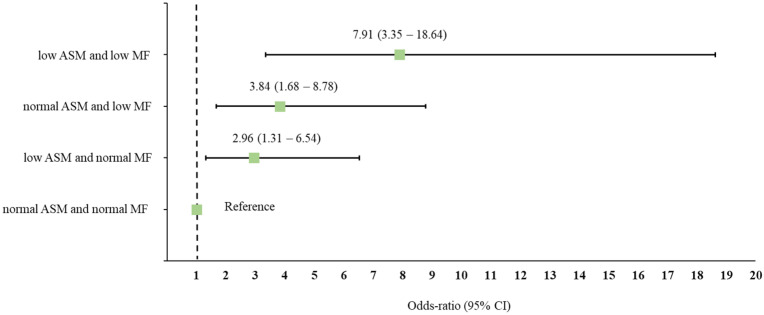
Odds ratio (OR) and 95% confidence interval (CI) for depressive symptoms according to appendicular muscle mass (ASM)- and muscle function (MF)-based subgroups. ORs were adjusted for covariates, including age, education, body mass index, waist circumference, medications, alcohol intake, smoking, physical activity, handgrip strength, and agility performance.

Figure 2 illustrates the risk of having depressive symptoms according to ASM- and MF-based subgroups. Individuals with low ASM/normal MF (OR = 2.963, 95% CI = 1.318–6.538, *p =* 0.019), normal ASM/low MF (OR = 3.843, 95% CI = 1.679–8.797, *p =* 0.002), and low ASM/low MF (OR = 7.907, 95% CI = 3.354–18.640, *p* < 0.001) had a higher risk of depressive symptoms compared to individuals with normal ASM/normal MF (OR = 1).

**Figure 3:**
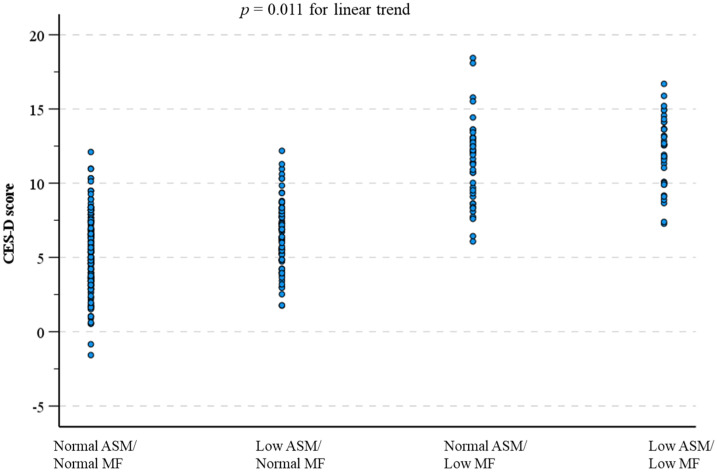
Depressive symptoms stratified by low appendicular muscle mass (ASM)- and low muscle function (MF)-based subgroups. CES-D: the 20-item Center for Epidemiologic Studies Depression Scale.

Furthermore, CES-D score was incremental (*p* < 0.011) in the order of normal ASM/normal MF, low ASM/normal MF, normal ASM/low MF, and low ASM/low MF, respectively, as shown in Figure 3.

## 4. Discussion

In this study, we examined the relationship of ASM and MF with depressive symptoms in community-dwelling Korean older adults. The current findings of the study suggest that either low MF or the coexistence of low ASM and low MF may represent a substantial risk for depressive symptoms, with low MF playing a more dominant role in this relationship.

Overall, the current findings of the study are in accordance with previous studies that have reported a significant association between sarcopenia and depression in Asian geriatric populations [4,15,30]. Previous studies also showed that geriatric depression was significantly associated with indicators of muscle function, such as low gait speed [31] and low handgrip strength [32,33]. In a cross-sectional study of 432 urban-dwelling Japanese older adults, Hayashi et al. [34] examined the relationship between sarcopenia and depressive mood and found that low handgrip strength and low physical performance (but not muscle mass) were significant determinants of depressive mood.

Likewise, previous studies have also examined the association between low ASM and depression in Korean populations but have reported inconsistent findings. For example, the association was significant in the Ansan Geriatric (AGE) study [14] but not in the 2010–2011 Korean National Health and Nutrition Examination Survey (KNHNES) [15]. In contrast, Han et al. [35] showed that low handgrip strength was significantly associated with depressive symptoms in socially deprived Korean older adults. In a recent study involving 885 community-dwelling older adults, Park et al. [36] found that osteosarcopenia, which was defined as the presence of low bone mineral density in addition to low ASM and low MF, was significantly associated with health problems such as disability, frailty, and depression. Together, the findings from the current and previous studies have showed that loss of MF or, preferably, the coexistence of low ASM and low MF is a better predictor of depression in geriatric populations, supporting the use of the sarcopenia criteria recommended by the AWGS [6,19].

Several explanations are possible for the relationship between sarcopenia and the risk of depression. First, the age-related loss of ASM and MF may increase the propensity to experience depression/depressive symptoms, perhaps due to declines in physical activity and/or physical fitness. In support of this notion, the current findings of the study also showed that individuals who were at increased risk of depressive symptoms had low physical activity level and poor physical performance. In contrast, it is well known that physical activity and/or fitness promotion relieve depressive symptoms or mood or prevents or attenuates depression risk via its antidepressant effects [37]. Second, geriatric sarcopenia results in physical limitations, an increased risk of falls, increased dependence, and decreased social inclusion; these poor quality of life profiles may collectively increase the risk of depression as well as cognitive dysfunction [38,39]. Third, age-related low-grade inflammation and increased oxidative stress may also explain the relationship between sarcopenia and depressive symptoms [40,41].

The present study had several limitations. First, the cross-sectional nature of the study limits any determination about causality regarding the association between sarcopenia and depressive symptoms. Second, the relationship between sarcopenia and depressive symptoms may be influenced by other factors, such as nutritional status [42], socioeconomic status [31], and existing health conditions [43], which needs to be further addressed in a future study. Third, a reverse relationship between sarcopenia and depressive symptoms may also exist. That is, depression may trigger loss of muscle mass and/or muscle function via decreased physical activity and/or decreased physical fitness [44,45], which needs to be further addressed in a future study. The study has some strengths, too. First, quantification of ASM was based on DXA, which is known as a gold standard for body composition analysis. Second, calculated STS muscle power based on 30 s chair stand test was used as an indicator of MF, which is known as a reliable and valid index for functional capacity and mortality in a recent study involving older adults [23]. Third, to the best of our knowledge, this is the first attempt to examine the individual and synergistic relationships of low ASM and low MF with depressive symptoms in Korean adults.

## 5. Conclusions

In summary, this cross-sectional study examined the relationship of sarcopenia with depressive symptoms in community-dwelling Korean older adults. We found that either low MF only or the coexistence of low ASM and low MF was significantly associated with increased depressive symptoms. Yet, the current findings of the study remain to be confirmed in an intervention targeted at both muscle mass and function against geriatric depression and/or depressive symptoms. From a clinical perspective, the current findings of the study also suggest that clinicians should check elderly persons with sarcopenia as well as dynapenia for an early detection of mental illness such as depression.

## Figures and Tables

**Table 1:** Characteristics of study participants.

Variables	All (*n* = 521)	Men (*n* = 164)	Women (*n* = 357)	*p* Value
Age (years)	74.1 ± 6.2	74.0 ± 5.9	74.1 ± 6.2	0.933
BMI (kg/m^2^)	24.6 ± 3.1	24.0 ± 2.5	24.8 ± 3.2	0.035
Body fat (%)	34.2 ± 7.9	24.6 ± 6.4	36.6 ± 6.2	<0.001
WC (cm)	92.0 ± 13.6	93.9 ± 12.7	91.5 ± 13.8	0.149
ASM (kg)	14.7 ± 3.1	19.9 ± 2.4	13.4 ± 1.5	<0.001
Education (years)	7.3 ± 4.5	10.8 ± 4.5	6.4 ± 4.1	<0.001
Alcohol intake, *n* (%)				<0.001
0	69 (13.2)	22 (13.4)	47 (13.2)	
≤1	152 (29.2)	43 (26.2)	109 (30.5)	
≥2	53 (10.1)	45 (27.4)	8 (2.2	
Smoking, *n* (%)				<0.001
Never	293 (56.2)	77 (47.0)	216 (60.5)	
Past/current	59(11.3)	38 (23.1)	21 (5.9)	
Number of medications, n (%)				<0.001
0	52 (12.1)	25 (15.6)	38 (10.6)	
1	133 (25.5)	32 (19.5)	101 (28.3)	
2	123 (23.6)	49 (29.9)	74 (20.7)	
≥3	28 (5.4)	8 (4.9)	20 (5.6)	
Parameters of physical performance				
Handgrip strength (kg)	20.1 ± 7.5	30.3 ± 5.8	17.4 ± 5.2	<0.001
30 s STS test (reps)	14.4 ± 4.3	16.0 ± 4.5	14.0 ± 4.2	<0.001
STS mean power (W)	173.27 ± 69.84	248.18±82.99	154.59 ± 51.40	<0.001
relative STS power (W/kg)	2.92 ± 0.99	3.75 ± 1.14	2.71 ± 0.83	<0.001
Physical activity (METs/week)	1059 ± 1217	1580 ± 1455	971 ± 1117	<0.001
MMSE-DS (score)	25.3 ± 3.7	26.9 ± 2.5	24.9 ± 3.8	<0.001
CES-D (score)	6.2 ± 8.6	5.3 ± 7.4	6.5 ± 8.9	0.265
Depressive symptoms, *n* (%)	111 (21.3)	34 (20.7)	77 (21.5)	0.644

BMI: body mass index, ASM: appendicular skeletal muscle mass, WC: waist circumference, STS: sit-to-stand, CES-D: the Center for Epidemiologic Studies Depression, MMSE-DS: Mini-Mental State Examination of Dementia Screening.

**Table 2:** Comparison of outcome variables between appendicular skeletal muscle mass (ASM)- and muscle function (MF)-based subgroups.

Variables	Normal ASM/Normal MF (*n* = 297)	Low ASM/Normal MF (*n* = 109)	Normal ASM/Low MF (*n* = 64)	Low ASM/Low MF (*n* = 51)	*p* Value for Group
Age (years)	73.3 ± 5.9 ^c,d^	73.8 ± 502 ^c,d^	78.1 ± 5.7 ^a,b^	78.2 ± 6.0 ^a,b^	<0.001
Education (years)	7.9 ± 4.5 ^c,d^	8.3 ± 4.0 ^c,d^	4.2 ± 3.4 ^a,b^	5.1 ± 4.7 ^a,b^	<0.001
Body mass index (kg/m^2^)	25.2 ± 3.0 ^b,d^	22.8 ± 3.1 ^a,c^	25.5 ± 2.5 ^b,d^	23.7 ± 2.6 ^a,c^	<0.001
Obesity ≥ 25kg/m^2^ (*n*, %)	152 (51.2)	20 (18.3)	37 (57.8)	19 (37.3)	<0.001
Waist circumference (cm)	92.1 ± 13.0 ^c^	90.2 ± 13.3 ^c,d^	95.9 ± 14.0 ^a,b^	95.3 ± 15.9 ^b^	0.007
Abdominal obesity (n, %)	183 (61.6)	58 (53.2)	53 (82.8)	34 (66.7)	0.001
Body fat (%)	34.2 ± 7.9	33.1 ± 8.2^c^	35.7 ± 6.6^b^	35.0 ± 8.7	0.227
Systolic BP (mmHg)	128.2 ± 14.1	128.6 ± 14.4	130.8 ± 13.4	132.4 ± 13.1	0.624
Diastolic BP (mmHg)	71.0 ± 8.7	71.5 ± 9.5	71.7 ± 9.5	71.8 ± 10.5	0.948
Hypertension (*n*, %)	52 (17.5)	22 (20.2)	14 (21.9)	12 (23.5)	0.820
Physical activity (METs/week)	1191.1 ± 1267.9	1092.1 ± 1269.1	1035.7 ± 1253.8	725.3 ± 622.8	0.126
Hand grip strength (kg)	22.2 ± 7.0 ^c,d^	21.1 ± 6.9 ^c,d^	17.1 ± 6.7 ^a,b^	16.8 ± 4.0 ^a,b^	<0.001
Time up-and-go test (s/3 m)	6.83 ± 1.55 ^c,d^	6.94 ± 1.82 ^c,d^	8.75 ± 3.02 ^a,b^	8.94 ± 1.87 ^a,b^	<0.001
30 s sit-to-stand (times/sec)	15.7 ± 3.8 ^c,d^	15.3 ± 3.4 ^c,d^	9.4 ± 1.7 ^a,b^	9.3 ± 1.7 ^a,b^	<0.001
STS mean power (W)	197.98 ± 68.96 ^b,c,d^	177.38 ± 51.92 ^a,c,d^	104.83 ± 29.28 ^a,b^	96.80 ± 20.14 ^a,b^	<0.001
relative STS power (W/kg)	3.23 ± 0.92 ^c,d^	3.18 ± 0.72 ^c,d^	1.80 ± 0.35 ^a,b^	1.76 ± 0.34 ^a,b^	<0.001
CES-D score	4.6 ± 6.8 ^c,d^	6.0 ± 8.5 ^c,d^	12.6 ± 11.3 ^a,b^	14.1 ± 10.9 ^a,b^	<0.001
Depression symptoms (*n*, %)	32 (10.8)	21 (19.3)	27 (42.2)	31 (60.8)	<0.001
MMSE-DS score	26.4 ± 3.2 ^c,d^	26.1 ± 3.2 ^c,d^	22.5 ± 4.5 ^a,b,d^	22.1 ± 3.3 ^a,b,c^	<0.001
Cognitive impairment (*n*, %)	41 (13.8)	16 (14.7)	17 (26.6)	15 (29.4)	0.009

BP: blood pressure; STS: sit-to-stand; CES-D: the Center for Epidemiologic Studies Depression, MMSE-DS: Mini-Mental State Examination of Dementia Screening; different superscripts (i.e., ^a, b, c, d^) represent significant group differences in measured parameters by least significance difference (LSD) post hoc tests; obesity was defined as BMI of ≥25 kg/m^2^ [27]; abdominal obesity was defined as WC of ≥90cm for men and WC of ≥85cm for women [27]; hypertension was defined as resting SBP of ≥140 mmHg or higher, or resting DBP of ≥90 mmHg, or taking antihypertensive medications [28]; cognitive impairment was defined according to age- and education-specific cut-points for Koreans [29]; significantly different compared to ^a^ normal ASM and normal MF, ^b^ low ASM and normal MF, ^c^ normal ASM and low MF, and ^d^ low ASM and low MF.

## Data Availability

Data can be accessible upon request to corresponding author (hkang@skku.edu).
